# Genetically engineered *Streptomyces viridosporus* ATCC 14672 strains for the discovery of novel moenomycins

**DOI:** 10.1038/s41598-026-43988-6

**Published:** 2026-03-10

**Authors:** Bohdan Ostash, Roman Makitrynskyy, Mariana Fedchyshyn, Andriy Luzhetskyy, Suzanne Walker, Victor Fedorenko

**Affiliations:** 1 Department of Genetics and Biotechnology, Ivan Franko National University of Lviv, Hrushevskoho st. 4, Rm. 102, Lviv, 79005 Ukraine; 2 German-Ukrainian Core of Excellence in Natural Products Research (CENtR), Zelena st. 20, Lviv, 79005 Ukraine; 3 Leibniz Institute DSMZ-German Collection of Microorganisms and Cell Cultures GmbH, Inhoffenstraße 7B, 38124 Braunschweig, Germany; 4 Helmholtz Institute for Pharmaceutical Research Saarland (HIPS), UdS Campus, Bld. E8.1, 66123 Saarbrücken, Germany; 5 Department of Microbiology, Harvard Medical School, 4 Blackfan Circle, Boston, MA 02115 USA

**Keywords:** Antibiotics, Moenomycins, Genes, *Streptomyces*, Genetic engineering, Natural products, Biochemistry, Genetics, Microbiology, Molecular biology

## Abstract

*Streptomyces viridosporus* (formerly *ghanaensis*) ATCC14672 is the best-studied producer of moenomycins, a family of phosphoglycolipid natural products directly inhibiting peptidoglycan glycosyltransferases. The uniqueness of moenomycins, in terms of structure and mode of action, and their extreme potency against several Gram-positive pathogens (including vancomycin- and methicillin-resistant cocci), are their advantages as a drug candidate. However, they are not orally bioavailable; in the bloodstream, moenomycins are characterized by an exceedingly long half-life. These shortcomings are thought to be caused by the long C25 lipid chain (moenocinol) of moenomycins. Here we report the generation and initial studies of two ATCC 14672 mutants to access a greater diversity of moenomycins around its lipid chain. The dO5 mutant cannot produce any moenomycins due to the deletion of the *moeO5* gene for the first step in moenomycin assembly. The M12 mutant of ATCC 14672 (knockout of the prenyltransferase gene *moeN5*) accumulates moenomycins with a C15 lipid chain instead of moenocinol. We demonstrate and discuss the potential applications of the dO5 and M12 mutants for the discovery of novel moenomycins and their genetic determinants.

**Supplementary Information:**

The online version contains supplementary material available at 10.1038/s41598-026-43988-6.

## Introduction

**Fig. 1:**
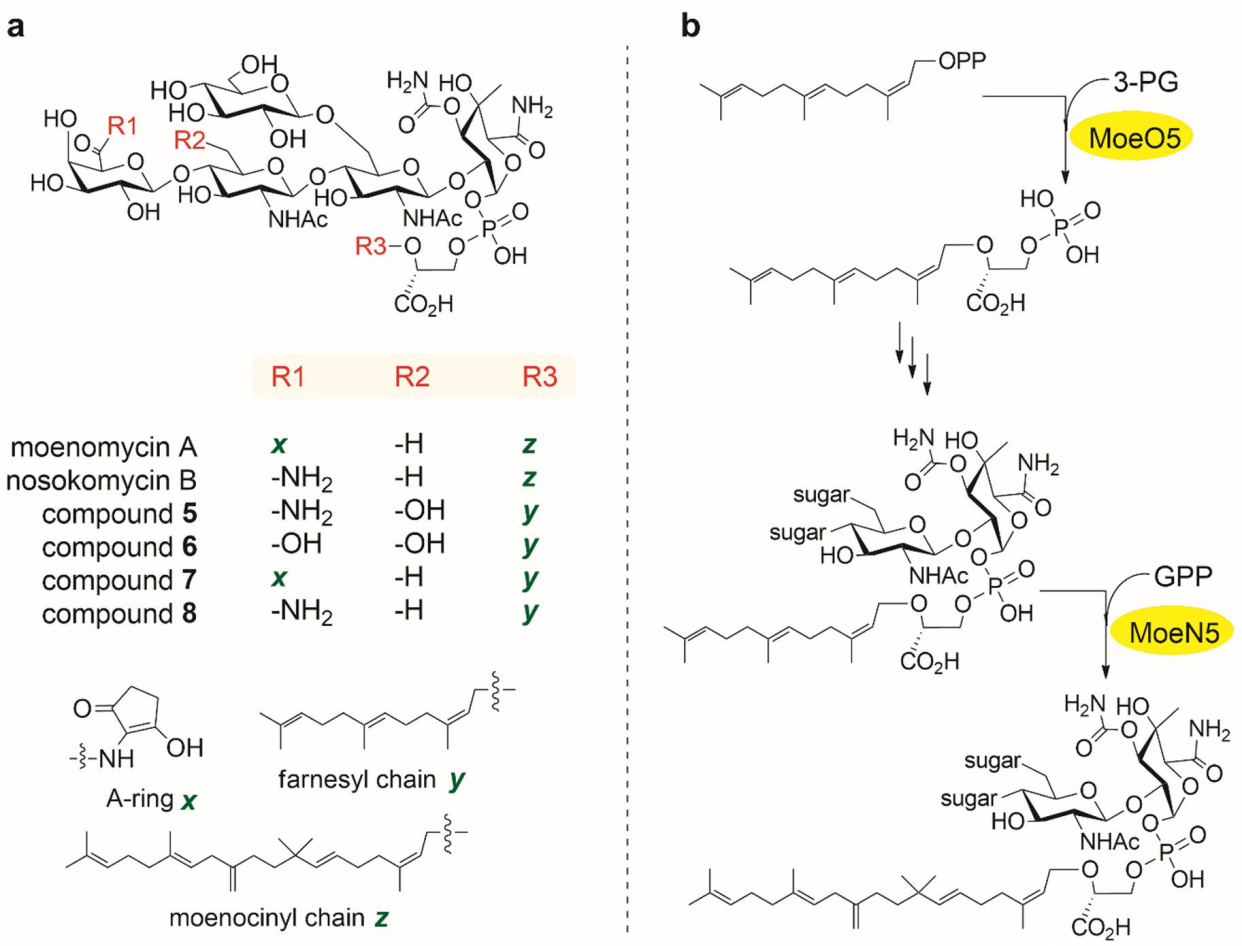
Chemical structures of the moenomycins mentioned in this work (**a**) and the reactions catalyzed by enzymes MoeO5 and MoeN5 (**b**).

The rise of resistance to common antibiotics over the last decade has resulted in reduced livelihood, lost lives, and an increased healthcare burden^1^. This is a worldwide problem that has to be countered via the development of new classes of antibiotics. Moenomycins are a small family of phosphoglycolipid natural products with several remarkable features from a drug development point of view, including a broad spectrum of activity and a unique mode of action^2^. Particularly, the archetypal member of the family, moenomycin A (MmA, Fig. 1a), is the only known direct inhibitor of peptidoglycan glycosyltransferases at concentrations in the nanomolar range^3,4^. It has been used in animal nutrition for four decades, with no significant resistance observed. However, MmA has an exceedingly long half-life and is not orally bioavailable, preventing its clinical use^3^. The chemical synthesis of MmA analogs/fragments is a challenging task^5–8^, which motivates the search for more efficient tools to explore the chemical space around the MmA scaffold.

We identified the MmA biosynthetic pathway in *Streptomyces viridosporus* (*ghanaensis*) ATCC14672 and studied MmA action in detail^2,9–11^. Our motivation behind this effort was that genetic engineering of the MmA producer ATCC 14672 could be an efficient way of discovering and producing novel, structurally complex phosphoglycolipids for biological testing and/or chemical modification^9^. To this end, many moenomycin analogs with trimmed olisaccharide and/or lipid portions were generated^7,10,11^; regulation of its production was elucidated^2,12,13^; and structures of several moenomycin biosynthetic enzymes were determined^14–16^. It is reasonable to focus on the diversification of the C25 isoprenyl (moenocinyl) lipid portion of MmA because of its relevance to pharmacokinetic issues. Current knowledge on this topic is detailed in^5^. Briefly, delipidated moenomycins, those with short lipid (C10 or less) and loss of charge/phosphate group on 3-phosphoglyceric unit display drastically diminished antibiotic properties; unit A (substituent *x* in Fig. 1a), which does not seem to contact the target^8^, augments to some extent antibiotic properties of moenomycins^10^.

The formation of moenocinyl (substituent *z* in Fig. 1a) is governed by two genes, *moeO5* and *moeN5*. Gene *moeO5* encodes the first dedicated enzyme in the moenomycin biosynthetic pathway^9^. The *moeN5* gene encodes a prenyltransferase that acts upon an oligosaccharide intermediate bearing a C15 (farnesyl) chain (Fig. 1b). We anticipated that a *moeN5* knockout in ATCC 14672 would lead to the accumulation of novel C15 moenomycins. The *moeO5*-deficient mutant would completely abrogate moenomycin biosynthesis. Different *moeN5* and *moeO5* homologs can be expressed in the aforementioned mutants, thus enabling the discovery of enzymes with novel substrate preferences and, hopefully, novel moenomycins. In this paper, we report the construction of *moeO5* and *moeN5* mutants of ATCC 14672 as well as their initial characterization.

## Materials and methods

### Strains and culture conditions

*Streptomyces viridosporus* ATCC 14672, *S. clavuligerus* ATCC 27,064, and *Bacillus cereus* ATCC 19,637 were obtained from the American Type Culture Collection (ATCC). *S. lividans* TK24 38 − 1+∆moeN5, described in^9^, was used to purify compound **5** (see Fig. 1). *Actinoplanes teichomyceticus* NRRL-B16726 was provided by ARS Culture Collection (NRRL). *E. coli* DH5α and BW25113 (pKD46), provided by J. Beckwith (Harvard Medical School), were used in routine cloning and recombineering experiments, respectively. *E. coli* ET12567 (pUZ8002), provided by C.P. Smith (Manchester University), was employed to conjugatively introduce plasmids and cosmids into ATCC 14672. *Staphylococcus aureus* 209P was a gift from Prof. Kozo Ochi (Hiroshima Institute of Technology, Japan). Tryptic soy broth (TSB; Merck cat. No 105459) was used to grow the bacteria for DNA isolation. Medium STSB (g/L: Difco tryptone peptone – 17, NaCl – 5, tapped water – to 1 L, pH 7.0 adjusted with 10 N NaOH) was used in the production of moenomycins. Oatmeal agar (OM) (10) was used to obtain sporulated actinomycete lawns. TSB agar was used for the bioassays and to check the absence of contamination in liquid actinomycete cultures. *E. coli* strains, ATCC 14672 and its derivatives, were cultivated at 37 °C unless otherwise stated. The other cultures were grown at 30 °C. Antibiotics were added to the growth media whenever necessary in the following concentrations: apramycin, 50 µg/mL; hygromycin, 100 µg/mL; kanamycin, 50 µg/mL; ampicillin, 100 µg/mL. A pure moenomycin sample was provided by Prof. D. Kahne (Harvard University).

### Molecular biology techniques, protein models and strain construction

The cosmid moeno38^10^ was used to prepare the *moeO5* and *moeN5* knockout constructs. Vectors pTES^17^ and pMKI9^11^ were used to construct the expression plasmids. Plasmids pIJ10700 and pIJ773 were used as sources of *oriT*-*hyg* and *oriT*-*aac(3)IV* cassettes, respectively^18^. The plasmid for *moeO5* expression in *Streptomyces*, pED2, was described previously^11^. All primers mentioned in this work are given in Table S1, Electronic Supplementary Materials (ESM). The codon-optimized synthetic gene *plu3366*, cloned into vector pUC57-kan, was ordered from Genewiz (South Plainfield, NJ); the nucleotide sequences of the natural and synthetic genes are given in Fig. S1, ESM. All constructs and mutants were verified by sequencing. Semi-quantitative RT-PCR analysis in ATCC14672 was carried out as described in^13^. AlphaFold Protein Structure Database (https://alphafold.ebi.ac.uk/) was used to compare structures of Plu3366 (Q7N1V3) and TchO5 (A0A1B1ESM3) to MoeO5 (PDB accession: 3VKA). Sequence similarity network analysis of Plu3366 was performed using EFI-Enzyme Similarity Tool (EFI-EST) (https://efi.igb.illinois.edu/efi-est*)*, with an edge selection cutoff of sequence identity > 40%, corresponding to an alignment score of 100^19^. Results from EFI-EST were visualized using Cytoscape 3.10.4 application, default parameters^20^. Phylogenetic analysis was run on server https://www.phylogeny.fr/index.cgi.

*Knockout of* *moeO5*. The primers moeO5-red-up and moeO5-red-rp were used to amplify the hygromycin resistance *oriT*-*hyg* cassette from pIJ10700, appending 40 bp sequences homologous to the flanks of *moeO5*. The cassette was gel-purified, digested with DpnI and used for the electroporation of arabinose-induced cells of *E. coli* BW25113 (pKD46) harboring the cosmid moeno38. Analysis of the resulting hygromycin- and kanamycin-resistant clones led to identification of cosmid moeno38dO5-Hy, in which the *moeO5* coding sequence had been replaced with *oriT*-*hyg*. The cosmid was then transferred conjugally from *E. coli* ET12567 (pUZ8002) into ATCC 14672, and the double crossover was selected based on hygromycin resistance (Hy^r^) and kanamycin susceptibility (Km^s^), as described in^10^. This resulted in generation of the ∆*moeO5*::*hyg* knockout strain dO5. The expected replacement in the cosmid and dO5 genome was confirmed by sequencing using the primers X5HindIIIup and moeN5EcoRIrp.

*Knockout of* *moeN5*. The generation of the cosmid and the ∆*moeN5*::*aac* knockout strain, M12, followed the pipeline described above for the *moeO5* gene. For this purpose, primers moeN5_red_for and moeN5_red_rev and the apramycin resistance *oriT*-*aac(3)IV* cassette from pIJ773, were used. Primers moeN5-d1 and moeN5-d2 were then used to sequence the knockout locus. *Plasmid pOOB78a*. The gene *SCLAV_p1286* (the *moeO5* homologue) was amplified with the primers moeO5c_XbaI and moeN5c_EcoRI from the *S. clavuligerus* ATCC27064 genome. The 0.9-kb amplicon was treated with the restriction endonucleases XbaI and EcoRI, then cloned into the respective sites of pMKI9 to create pOOB78a. There, transcription of SCLAV_p1286 is controlled by the strong *ermEp* promoter. *Plasmid pTEStchO5*. The gene *tchO5* (0.9 kb) was amplified with the primers tchO5_XbaI_up and tchO5_EcoRI_rp from the *A. teichomyceticus* genome. The gene was cloned as an XbaI-EcoRI fragment into the respective sites of pTES to give pTEStchO5. *Plasmid pOOB97a*. The codon-optimized *moeO5* homolog from *Photorhabdus luminescens*, *plu3366* (Fig. S1A, ESM), was retrieved from pUC57-kan as an XbaI-EcoRI fragment and cloned into the same sites of pTES to result in pOOB97a.

### Assays of moenomycin production and activity

Production of moenomycins under submerged conditions and their small-scale purification were performed as follows. A 100-mL flask containing 12 mL of TSB and 20 glass beads (Sigma; Ø 5 mm) was inoculated with an agar slice (approx. 2 sq. cm) cut off the lawn of *S. viridosporus* strain grown on OM for 168 h. This pre-culture was incubated on an orbital shaker (200 rpm) for 48 h. One mL of the pre-culture was transferred into the main 300-mL culture flask containing 35 mL of STSB and 40 glass beads. The main culture was then grown for 120 h under the conditions used to prepare the pre-culture. 30 mL of the main culture were transferred into 50-mL tubes, spun down and the supernatant was discarded. The cell pellet was resuspended in 20 mL of deionized water, spun down, and extracted in a mixture of 0.6 mL of deionized water and 7 mL of methanol (shaker at 200 rpm for 4 h at 37 °C). The extraction step was repeated one more time. The first and second extracts were combined and evaporated. The solid residue was reconstituted in 1 mL H_2_O – 0.1% NH_4_OH. A C18 SPE column (100 mg; Alltech) was activated via successive washes with methanol and water (1 mL each) and then the extract was loaded. The column was washed with water (0.5 mL), followed by 1 mL ethyl acetate, after which moenomycins were eluted with 1.2 mL of methanol. The eluate was evaporated and the dry residue was reconstituted in 50 µL of deionized water. The purification of moenomycins from the large-scale (0.5–1 L) submerged fermentation followed the procedure outlined in^9^. The purified extracts were used in agar plug, antibiotic disc diffusion and biochromatography assays, and LC-MS/MS experiments as described in^11^. Minimal inhibitory concentrations (MIC) were determined as described in^10^. An Agilent 6520 Q-TOF was used to acquire the accurate masses of the compounds and MS/MS spectra of the moenomycins (column Gemini C18 4.6 × 250 mm, 3 µM particle size).

## Results and discussion

### The *moeO5* knockout derivative of ATCC14672, dO5, as a tool to probe *moeO5* homologs

We replaced the *moeO5* gene within the ATCC14672 genome with a hygromycin resistance cassette, yielding the strain dO5. Sequencing of the dO5 genome from the primers encompassing the *moeN5*-*moeX5* locus confirmed the expected genetic rearrangement (Fig. S2, ESM). The dO5 strain did not produce moenomycins, as judged from LC-MS analysis (Fig. S3, ESM). In bioassays, dO5 cells and whole methanol extracts displayed very low, but yet still detectable, activity against *B. cereus* (Fig. S4a, b), which is likely due to the production of other antibiotics, some of which are known to be produced by this species^21–23^. Purified methanol extracts were devoid of antibiotic activity, providing simple readout of moenomycin production (Fig. S4c, d).

**Fig. 2:**
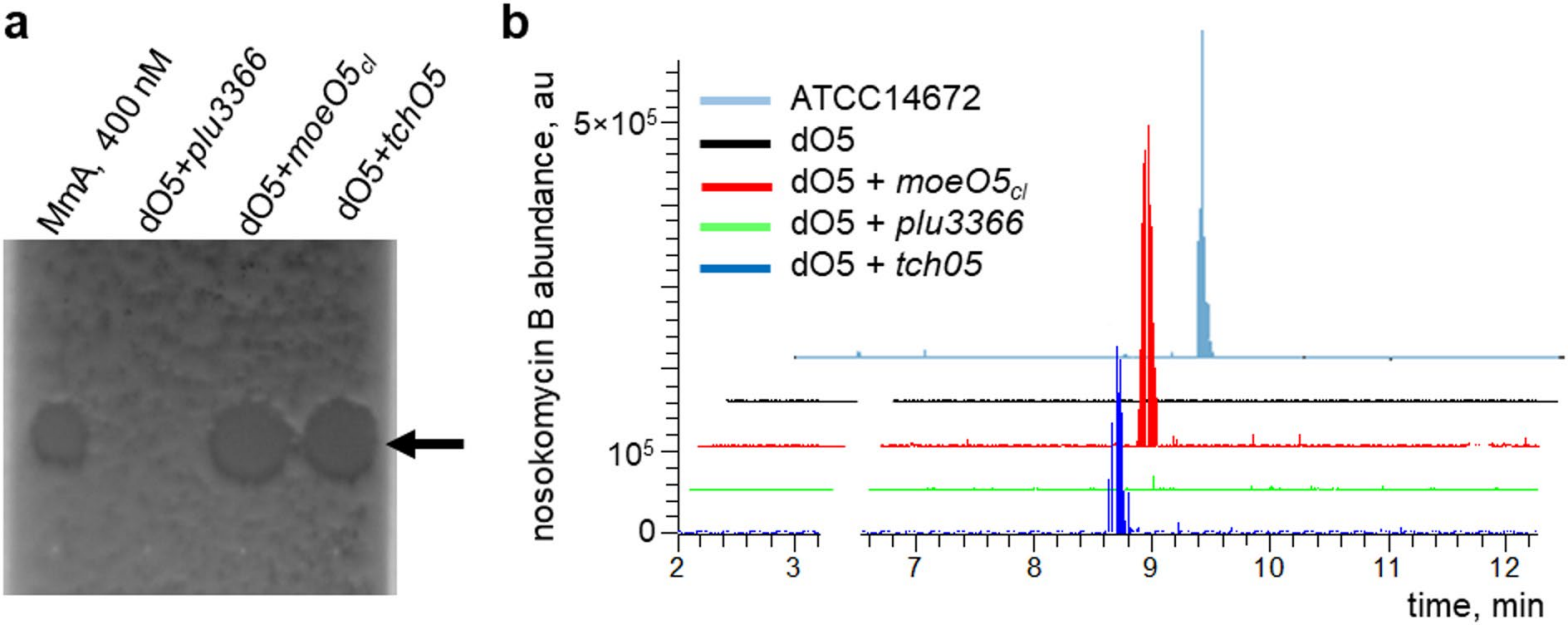
Probing the function of *moeO5* homologs via their expression in dO5 strain. Biochromatogram (**a**) of the purified extracts from the strains expressing various *moeO5* homologs. Black arrow marks bioactive spots corresponding to moenomycins. The leftmost lane is pure MmA sample. The stacked extracted ion chromatogram traces (**b**) correspond to the major moenomycin congener, nosokomycin B (1484.6 Da, [M-H]^−^; see Fig. 1 for the structure). No moenomycin-related mass-peaks (see Fig. S3) were found in dO5 or in dO5 harboring the *plu3366* expression plasmid. MS traces represent the typical results of three biological repeats.

As a proof-of-principle demonstration of the use of the dO5 strain, we have chosen three *moeO5* homologues. These were *SCLAV_p1286* (*moeO5*_*cl*_) and *tchO5* from moenomycin biosynthetic gene clusters of *Streptomyces clavuligerus* ATCC 27,064 and *Actinoplanes teichomyceticus* NRRL-B16726, respectively. The third, *plu3366*, originates from an obscure gene cluster within the genome of the entomopathogenic enterobacterium *Photorhabdus luminescens*. The *moeO5-* and *plu3366*-containing gene clusters are only distantly related, and the natural product(s) encoded by the latter remains unknown^5,24^. The aforementioned MoeO5 homologues display differing degrees of divergence of amino acid sequence with regard to MoeO5 from ATCC14672. MoeO5_cl_ and TchO5 share 73% and 67% similarity with MoeO5, respectively, while Plu3366 and MoeO5 are 46% similar. The multiple sequences alignment of these proteins (see Fig. S1, ESM) demonstrated a presence of almost all conserved residues contacting pyrophosphate and farnesyl-3-phosphoglycerate^13^. We constructed plasmids for the expression of these genes in *Streptomyces*, and in case of *plu3366* used a *Streptomyces* codon-optimized sequence (see Materials and Methods). The plasmids were introduced individually into dO5, and methanol extracts from the resulting strains and ATCC14672 were subjected to bioTLC and LC-MS analysis. Both MoeO5_cl_ and TchO5 restored moenomycin production to dO5; however, the *plu3366*-carrying derivative showed no antibacterial activity (Fig. 2a) and did not differ in its mass spectra from dO5 (Fig. 2b).

**Fig. 3:**
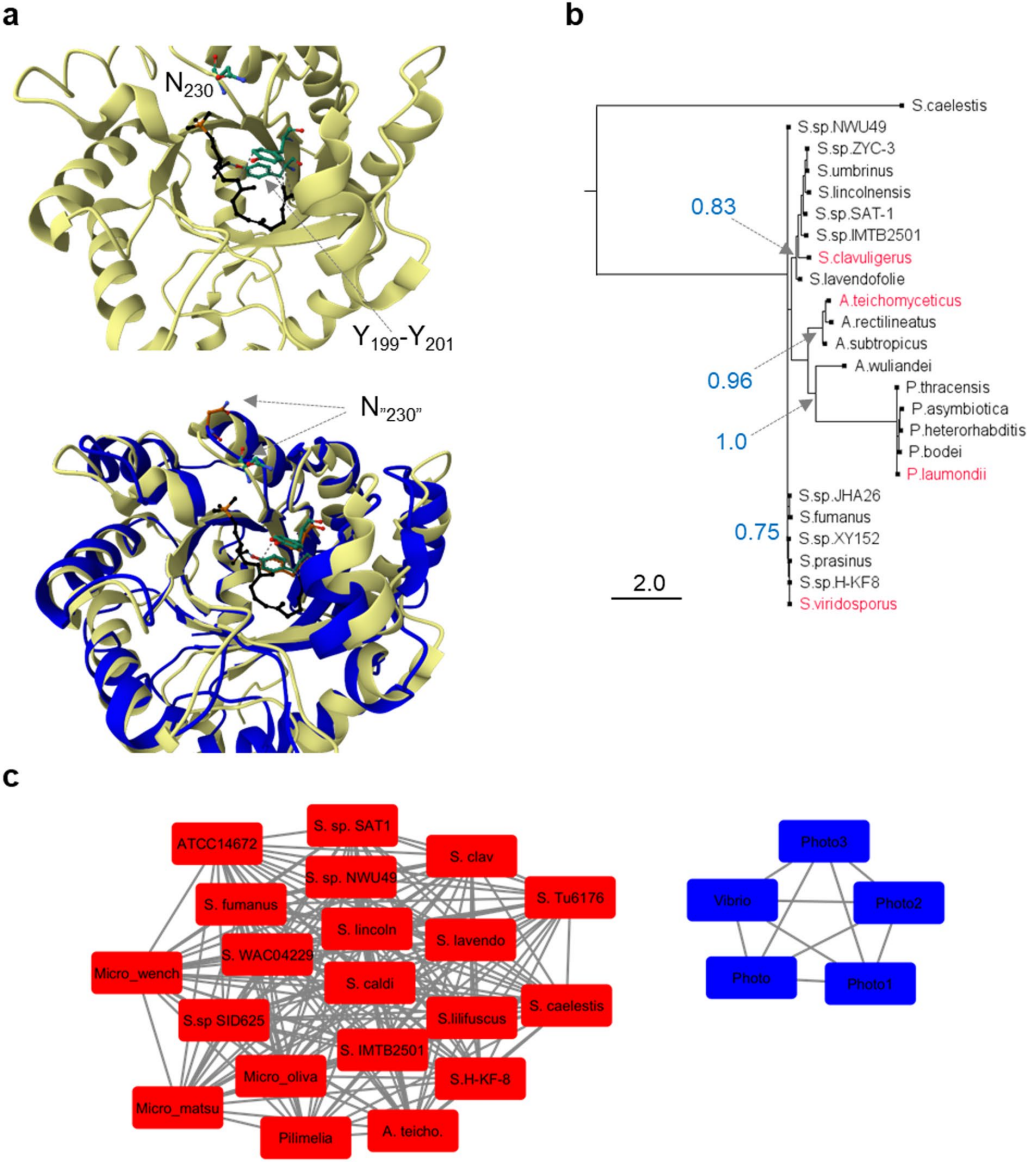
In silico insights into Plu3366 structure and function. 3D structure of MoeO5 (light yellow, part **a**, top) zoomed to the location of MoeO5 substrate (black ball and stick model with orange phosphorus atom). Conserved Tyr199, Tyr201, N230 are marked with arrows. MoeO5-Plu3366 superposition (part **a**, bottom) reveals that Tyr199-Tyr201 motif occupies the same spatial position in both proteins. Asn230 position in MoeO5 corresponds to Gly204 in Plu3366 model. A distally located Asn207 is present in Plu3366 (marked with arrows and labeled N_”230”_). See Fig. S6, ESM for MoeO5-TchO5 comparison. In maximum-likelihood protein tree (**b**) all MoeO5 homologs from *Photorhabdus* are a distinct subclade within the clade harboring sequences from *Actinoplanes* and *Actinomyces*. Numbers in blue denote the reliability of tree topology (aLRT indices) at a few key nodes (marked with arrows). Sequence similarity analysis (**c**), initiated with Plu3366 as a query, revealed 25 proteins forming two clusters: one for *Photorhabdus* and *Vibrio* proteins (blue), and another for “actinomycete” cluster (red). Abbreviations and protein accession numbers for SSN, see Fig. S6, ESM.

The inability of the *plu3366*-harboring strain to produce moenomycins was also supported by the bioassay results of concentrated extracts (Fig. S5A, ESM). Hence, while revealing the MoeO5 activity through the expression of an actinomycete gene in dO5 appears to be a straightforward task, the failure to complement the *moeO5* deletion with *plu3366* may have numerous explanations. The gene *plu3366* is transcribed just as well as the housekeeping gene *hrdB* is in ATCC14672 (Fig. S5B, ESM). Yet, currently we could not rule out expression roadblocks at the translation level/protein stability, as well as more fundamental issues with Plu3366 substrate preferences and/or downstream modification of Plu3366 product. If Plu3366 accepts substrates different from 3-phosphoglycerate and farnesyl pyrophosphate, this could derail the subsequent steps in moenomycin production. Comparison of X-ray structure of MoeO5-product co-complex^14^ with AlphaFold-predicted model of Plu3366 revealed their overall similarity and presence of conserved Tyr199-Tyr201 residues thought to form a characteristic “nook” for highly specific positioning of farnesyl chain within the catalytic pocket. There were also some differences: phosphate-contacting Asn230 in MoeO5 is replaced with glycine in Plu3366; an asparagine residue is located somewhat distally (Fig. 3a). This might compromise the ability of Plu3366 to contact phosphate group of the presumed substrate. Nevertheless, we note that similar situation is observed in case of TchO5 (Fig. S6, ESM). Plu3366 and its enterobacterial counterparts form a compact group in phylogenetic and sequence similarity network (SSN) analysis (Fig. 3b, c), distinct from actinomycete homologs. In the SSN, Plu3366 cluster showed no connections to the other proteins at 40% similarity cutoff value.

### The *moeN5* knockout ATCC14672 strain M12 yields novel C15-moenomycins

The gene *moeN5* was replaced with an apramycin resistance cassette, and the resulting mutant, M12 (∆*moeN5*::*aac(3)-oriT*), was verified as described in Methods. Phenotypically, M12 did not differ from the parental strain, but its antibiotic activity was severely reduced (Fig. S7, ESM), as would be expected for compounds with a shorter lipid chain^5,9^.

**Fig. 4:**
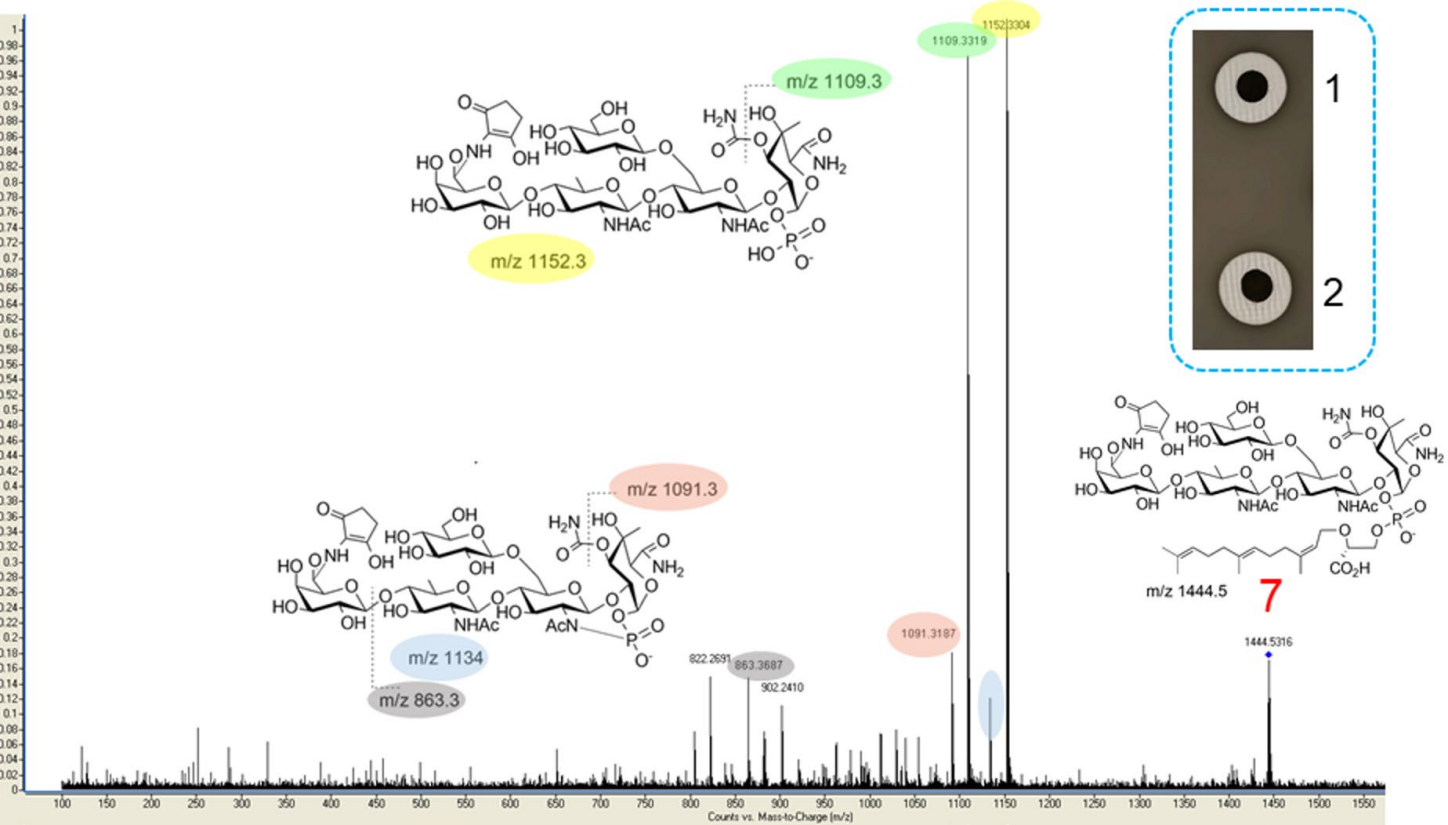
Collision-induced dissociation spectra for the compound **7** and testing its activity in an agar plug assay (blue line boxed inset, top right corner). The amount of the compound applied to the disk is as follows: 1, 800 nM of **7**; 2, 32 nM of MmA. The photo represents typical result of four independent experiments.

We took advantage of the extremely well-established MS fragmentation patterns for moenomycins^9,25^ to confirm that M12 indeed produced C15-moenomycins. In particular, the mutant accumulated compounds **7** and **8** (see Fig. 1), which are farnesyl-based analogs of MmA and nosokomycin B, respectively (Table 1). MS/MS analysis and disk diffusion assay for the major compound **7** is given in Fig. 4; MS/MS spectra of **8** is in Fig. S8, ESM.

**Table 1:** High-resolution MS data of the identified compounds.

Compound	Chemical formula	Retention time, min	m/z, [M-H]^-^		Mass error, ppm
			Calculated	Observed	
Compound 7	C_59_H_91_N_5_O_34_P_1_^-^	6.2	1444.5288	1444.5335	4.3
Compound 8	C_54_H_87_N_5_O_32_P_1_^-^	6.5	1348.5077	1348.5110	3.8

We were not able to detect the accumulation of glycine-bearing (moenomycin G) analogs with a C15 lipid. Thus, the compounds **7** and **8** broaden the chemical space of moenomycins with shorter lipid chain accessible via biosynthesis. In contrast to heterologously produced^9^ compounds **5** and **6** (see Fig. 1), compound **7** features a C5N unit, known to increase the biological activity of moenomycins^5,10^. This also demonstrates that the amidotransferase MoeH5, responsible for transferring the C5N unit to the terminal galacturonate^10^, recognizes moenomycins with either C25 or C15 lipids as its substrates.

We purified compounds **5** (from *S. lividans* 38 − 1^+^∆*moeN5*^7^ and **7** (from *S. viridosporus* M12) from 4 L of fermentation broth, with a yield of about 0.1 mg L^− 1^; the purity was over 90%, as determined by TLC, UV measurements and LC-MS (Fig. S9). The antibiotic activity of these compounds was compared to MmA. As judged from the disk diffusion assay (see Fig. 4), **7** is perhaps by more than one order of magnitude less active than MmA. Given the inherent shortcomings of disk diffusion assay for compounds with different physical-chemical properties (in our case, different lipid chains), we determined minimal inhibitory concentrations (MIC) for **5**, **7** and MmA against *Staphylococcus aureus* 209P. These data, presented in Table 2, demonstrate a reduction, by over two orders of magnitude, in activity of compound **5** featuring farnesyl chain and absence of C5N unit, as compared to MmA. Presence of C5N unit (compound **7**) recovered the activity, although this gain is still within one order of magnitude as compared to **5**, thus far below the potency of MmA.

**Table 2:** MIC values (µg mL^− 1^)* of selected moenomycins against *S. aureus* 209P.

	Moenomycins		
	MmA	5	7
*S. aureus* 209P	0.09	45.0	8.0

*Average values of 10 measurements.

## Conclusion

Diversification of the moenomycin scaffold around the lipid chain is desirable to make it more attractive for drug development purposes. With this in mind, we have generated two *S. viridosporus* ATCC 14672 mutants that are deficient in the assembly of the C25 moenocinyl chain. The *moeN5* null strain M12 produces two novel moenomycins with a C15 chain, as evident from MS/MS data and altered chromatographic properties. The *moeO5* null strain dO5 is completely derailed in moenomycin production. While M12 serves as a source of novel phosphoglycolipid products, both mutants are useful for discovering novel moenomycin biosynthetic genes. The availability of the latter has increased dramatically thanks to the ever-increasing pace of bacterial genome sequencing and the analytical power of genome mining tools^26,27^. Still, the bottleneck is our ability to functionally interrogate such novel genes. The described here strains are therefore a platform for expressing *moeO5* and *moeN5* homologues (either found in other bacteria, or derived through protein engineering), whose ability to catalyze the production of a first dedicated intermediate, suitable for downstream conversion into antibiotically active compounds, can be quickly detected using a combination of bioassay and mass spectrometry approaches. While MoeO5 counterparts unable to complement *moeO5* loss in dO5 are not immediately useful for moenomycin diversification, their further biochemical study may unearth valuable properties that eventually could be transplanted into MoeO5. Finally, it is shown for the first time that moenomycins with C15 lipid chain have drastically reduced antibiotic activity, which can be to a certain degree improved upon a modification of distal carbohydrate part.

## Supplementary Information

Below is the link to the electronic supplementary material.

Supplementary Material 1

## Data Availability

Data is provided within the manuscript and supplementary information files.
