# Supplementary material for: Genetically engineered Streptomyces viridosporus ATCC 14672 strains for the discovery of novel moenomycins
Source: Sci Rep. 2026 Mar 10;16:12851. doi: 10.1038/s41598-026-43988-6 (PMC13096315; doi:10.1038/s41598-026-43988-6)
Supplement: Supplementary file 1 — Supplementary Material 1 [file 41598_2026_43988_MOESM1_ESM.pdf]

Electronic Supplementary Materials to the paper of Ostash B. et al. Genetically engineered *Streptomyces viridosporus* ATCC14672 strains for the discovery of novel moenomycins

Inventory of supplemental tables and figures

Table S1. Primers used in this work
Figure S1. Nucleotide sequences of *plu3366* and its *Streptomyces*-optimized version *a-plu3366*
Figure S2. Knockout of *moeO5*.
Figure S3. Mutant dO5 does not produce moenomycins
Figure S4. Bioassays of *S. viridosporus* strains as a facile readout of moenomycin production.
Figure S5. Bioassays of dO5 expressing *moeO5* homologs and RT-PCR analysis of *plu3366* transcription in dO5 strain
Figure S6. Input data for SSN analysis and comparison of TchO5 model with MoeO5-product co-complex
Figure S7. Phenotype and biological activity of *moeN5*-deficient strain M12
Figure S8. CID spectra of compound **8**
Figure S9. Purification of compounds **7** and **5**
Figure S10. Uncropped, unedited images for the Fig. 3, Fig S4
Figure S11. Uncropped image of the gel shown in the Fig S5
Supplementary References

Table S1. Primers used in this work

Primer name	Sequence, 5´ → 3´	Purpose
moeO5c_XbaIup	AAATCTAGAGGACTGTCATGGCACCGTTC	To clone *moeO5* homologue from *S. clavuligerus* into vector pMKI9.
moeN5c_EcoRIrp	AAAGAATTCAGAGCCCCAGCTCCGCG	
moeO5-red-up	GTGAACGCCTCACCGCAACTGGACCACCAC ACGGAACTCATTCCGGGGATCCGTCGACC	*oriT-hyg* cassette with 40-bp homologous arms on the flanks of *moeO5*
moeO5-red-rp	CAGGTACACCATGTGGAAACCGAAGGCACG GGCGACGTGTGTAGGCTGGAGCTGCTTC	
X5HindIIIup	AAAAGGCCTGCCACCCTGCGGATGGCG	Primers used to amplify *moeN5-moeX5* region of *moe* cluster to verify *moeO5* replacement
moeN5EcoRIrp	AAAGAATTCCGCTGATCAACACGTCGCTC	
moeN5_red_for	ATTCCGGGGATCCGTCGACCATGCTCGCCG CCGAGGCCGCCAACCGCGACCATGTCACG	*oriT-aac(3)IV* cassette with 40-bp homologous arms on the flanks of *moeN5*
moeN5_red_rev	TGTAGGCTGGAGCTGCTTCTCACTCCCCCA GGTGCCGGGGAAGCAGCCGTACCAGCAC	
moeN5-d1	CGGCTGTACGGTCTGCTCGC	Diagnostic primers to verify *moeN5* replacement
moeN5-d2	GAGCCCTGGAACAGCCGGAC	
tchO5_XbaIup	AAATCTAGACCGGACAGCTACGAGGACTG	To clone *tchO5* from *A. teichomyceticus* into vector pTES
tchO5_EcoRIrp	AAAGAATTCGGCATGCCAATCCGATCAGG	
hyg_diagn11	GGTCGGTCGTGCCGTCCATC	To verify moeO5 knockout
rt_hrdB_ghana_up	GAGTTCGGCGACCTCATTG	RT-PCR analysis of ATCC14672 genes (420 bp)
rt_hrdB_ghana_rp	CGTCTTGGACTCGATCTGG	
rt_plu3366_up	GACGCGTTCTTCGCCACGAG	RT-PCR analysis of synthetic *plu3366* gene within ATCC14672 (405 bp)
rt_plu3366_rp	CGAAGAGCAGCGTGTTGTCG	

A

Original nucleotide sequence of *plu3366* of *Photorhabdus luminescens* subsp. *laumondii* TTO1

```
ATGGATATTTGTAAATATATGTCTTCATGTAGAAAGTCAATAATCCCTATCCTCGATCCATTTAAGTTTAATTA
TGAAATAAATATAAATAGATTATTAACTTTTCAAAAATATGCTCCTTTTTGTATAATAGCTAGTACGGATTGTG
AAAACTTTGAGATGAAAGTGTCTCCCTTTATAGCTGCCGCATCAAAGATTAAAACCATCCCGATCATAACGCAT
TTTCCTCCTCAGAAACCAAACGGATTTCCTTCATCACCTCACGCAGATGCATTTTTTGCCACATCAGTGATAAA
TTCCAATGTGGAATACTATAGCTCTTTATCATTAGAGAGAGAGTCCATCAAGCAGAGTATTGAAAAGTATGGTG
ACAACTGTTTAATCAGTTCTGCTGCTCTTGTTCTAGGTAATGATAAAAAGTCTCAAAAATTTGTTTATTCAAAG
ACTGTAGATCACTCAACAGAAAGTATTGCTAGAGCATTGGAAGGTATAGATTTCAATTTATTGAAGGTTTTTTA
CCTTTACTCAAGAAACAGCGTTATTTCTAATGAAATATGCAGTTTTGTAAAGTTTATTGTTGGAGATAATACAC
TGTTGTTTGTTAGTGGAGGAATATCAAACACTGAGCAGGTAAATAACCTGTTATATTCTGGTGCTGATTTTATT
TCAATTGGAACTACATTTGAAGAAGTCAATTGGAATAACAATGCTATAAACATATTAAATAAAGAAAGAATATA
TTAA
```

Nucleotide sequence of *a-plu3366*, plu3366 optimized to *Streptomyces* codon usage and decorated with RBS and restriction sites

```
TCTAGAGCAACGGAGGTACGGACATGGACATCTGCAAGTACATGAGCTCGTGCCGGAAGTCGATCATCCCGATC
CTGGACCCCTTCAAGTTCAACTACGAGATCAACATCAACCGGCTGCTGACGTTCCAGAAGTACGCGCCGTTCTG
CATCATCGCCTCCACGGACTGCGAGAACTTCGAGATGAAGGTCTCCCCGTTCATCGCGGCCGCCTCCAAGATCA
AGACCATCCCGATCATCACGCACTTCCCGCCGCAGAAGCCCAACGGGTTCCCGTCGTCCCCGCACGCCGACGCG
TTCTTCGCCACGAGCGTCATCAACAGCAACGTGGAGTACTACTCCTCCCTGTCCCTCGAGCGGGAGTCCATCAA
GCAGTCCATCGAGAAGTACGGCGACAACTGCCTGATCAGCAGCGCCGCGCTGGTGCTCGGCAACGACAAGAAGA
GCCAGAAGTTCGTCTACTCGAAGACCGTCGACCACTCGACGGAGTCCATCGCCCGGGCGCTGGAGGGCATCGAC
TTCAACCTGCTCAAGGTGTTCTACCTGTACTCGCGCAACTCCGTCATCTCCAACGAGATCTGCTCCTTCGTGAA
GTTCATCGTGGGCGACAACACGCTGCTCTTCGTCAGCGGCGGCATCAGCAACACCGAACAGGTCAACAACCTCC
TGTACTCCGGGGCGGACTTCATCTCGATCGGCACCACGTTCGAGGAGGTCAACTGGAACAACAACGCGATCAAC
ATCCTCAACAAGGAGCGCATCTACTGAATTC
```

B

```
Plu3366         ------------------------MDICKYMSSCRKSIIPILDPFKFNYEININRLLTFQKYA-PFCIIASTDCENFEMKVSPFIAAASKIKTIPIITHF
TchmO5          MTTGEASMEATAEHDGPG---AKRRDVLAHLRAHRGHVIHVVDPFKVEVAEAVQKARAVTAAGMPALLLASTDYEDFETHMPAYLNAVRAATDIPTILHF
MoeO5           MNASPQLDHHTELHAAPP--LWRPGRVLARLREHQPGPVHIIDPFKVPVTEAVEKAAELTRLGFAAVLLASTDYESFESHMEPYVAAVKAATPLPVVLHF
SCLAV_MoeO5     MNAVPELTHRTPRKTTPPPAVFHPGKTLARLREHPPGPVHIIDPFKIPQHEAVEKAAALDALGFPAILLASTDYTAFEDRMEPYLAAIKQVTSLPLLLHF
                                                          *  *                        ***                         * 

Plu3366         PPQKPNGFPSSPHADAFFATSVINSNVEYY--SSLSLERESIKQSIEKYGDNCLISSAALVLGNDKKSQKFVYSKTVDHSTES----------------
TchmO5          PPIPGRGFPVVPEAESMMLPALLGSDDPYYVWKSLLETYALGNGVPPA---PQPLLSAALTFGRDDRSYARMGTRPVAQDEQS----------------
MoeO5           PPRPGAGFPVVRGADALLLPALLGSGDDYFVWKSFLETLAAFPGRIPREEWPELLLTVALTFGEDPRTGDLLGTVPVSTASTEE---------------
SCLAV_MoeO5     PPRKGIGFPLVAGADAVVLPALLGSTDDYYVWKSYLETLLALPVRLDREDWPELLLTVALTFGEDHKTGDLLGTVPVNTENIEDTENTEDSRGARGPAGG

Plu3366         --IARALEGIDFNLLKVFYLYSRNSVISNEICSFVKFIVGDNTLLFVSGGISNTEQVNNLLYSGADFISIGTTFEEVNWNNNAINILN----KERIY---
TchmO5          --VLAYAQRARQFGFDMVYLYSRNEQVTTRTCELFREVLAPEQLLFASGGVRTPEQVDAYLQAGADYVIFAGALETPDWRAALDRLCSAGQLQRRALSLT
MoeO5           --IDRYLHVARAFGFHMVYLYSRNEHVPPEVVRHFRKGLGPDQVLFVSGNVRSGRQVTEYLDSGADYVGFAGALEQPDWRSALAEIA--G--RRPAAPAR
SCLAV_MoeO5     NRLDTHIAVARDFGFHLVYLYSRYDRVPLDVIRRFRAGLRPGQILFVSGNVHRREQVDDYLAAGADCVGFAGALEHPDWRTTLKELADPG--RRRE----
                                  * * *                        *
```

Figure S1. Nucleotide sequence of optimized gene *a-plu3366* used to construct the expression plasmid pOOB97a (**A**) and (**B**) multiple sequence alignment (CLUSTALW) of MoeO5 homologs discussed in this work. Restriction sites are underlined, start and stop codons are highlighted with green and red background, respectively. In part B, green background denotes residues of MoeO5 involved in pyrophosphate coordination; yellow labels residues contacting farnesyl-3PG; H97 contacts carboxylate of 3PG via water molecule [1]. Asterisks mark residues conserved in all four proteins.

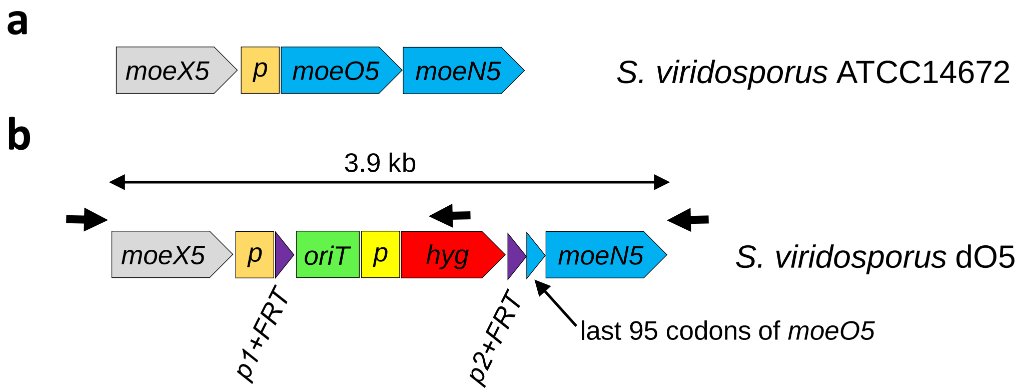

c

```
Sequence of moeX5-moeN5 stretch amplified from dO5 genome

ATGACGGCCACCCTGCGGATGGCGGAGATGACCTTCCGCGAACTGCTGCGCCGGCGGGGCGTGCTGGGCCTGC
TGCTCCTGGTCCCGCTCGTCTTCTACCTCGGGCGTTACGACCAGACCGGCCAGGCGGTCCGGTTCGCCAGCCT
CGGGGTGGGCTTCGCGGTCAGCGCCGCGGCCCTCTTCTCCGCGGTCGGCGGCCGGGAGATCGAACCGCTCCTG
GCCCTCTCCGGGTTCCGCCCGCTCCAGCTCTTCCTGGGCCGCCTGCTGGCCCTCCTCACCGCCGGCATGGGCG
TGTCCGCCCTCTACGCCGTGATCATCCTGGTCGGGCAGGACGTGGCGCACCCGCGGGCCGTCGCGGTGGAACT
GGCGCTGACCACACTGGTGGCGGTGCCGCTGGGACTGCTGCTCGGGGCGGCCGTGCCACGGGACATGGAGGGC
GCCCTGCTGCTGATCTCCGTCATCGGCGCCCAGATGGTGATGGATCCGGCCAAGGATTCGGCCAAGGTGCTTC
CCTTCTGGTCGACCCGGGAGATCATCACCTACGCGGTCGACGGCGCGGACAGCGGGTCGTTCGACTCCGGGGT
GGCCCACGCCGTCGGAGTGACGCTGCTGCTGGTCGCGGTGAGCGGTTGCGTGACGGCGGGCCGATTGCGCCGC
CGGCGCCATCTGCAATTCGCGTGAAATACGCCGGACACCGCCGAGAACGCCCCGCACGGCGCGAAACCGTCGG
GGCGTGCCTTCTTCCGTCCGGCAGGAATGCATAACCGCCGCACAAAGTCTCCCTACCCCACGGAAACGAACGC
GTGTAGTATCGGCAACCGCCCTGCCGCACCCTGTCCTCGAGGCGGCCCGGTGGACCCGCTGTTCGTCTCTCCA
GTGGAAGGGGAGTACGTGAACGACGCTTTTCGTCCGTCCGCCGGTGAATCCGCCGGACGGGCCCGCCGAAGGA
TTTCCGAGCACTTCCGCACTCCCTCGACCGCGGCGTGTGCCGGAACGGTCACGCAGTTCCCCGGGCGGGTCTG
AAGGAAACCGCGCGACGGCGCCGGACCACGGCTTTCGCGCCGGCGCGGCACACCGGGAGGAACACTCCCGGCC
GGCCGGGAACGGCCTCGGCGACCAGGGCGCCCCGCACCTCCATCCGAACGCGCGACACCTCATCCGGACGCGA
TCCGCACGTGCGACGCGGCCGGCACCCCGTCGCGTGACCCGACCGGACGACAAGCCGGACGACGAGCCGGACG
ACAAGGGGGAAGACGCTGGTGAACGCCTCACCGCAACTGGACCACCACACGGAACTCATTCCGGGGATCCGTC
GACCTGCAGTTCGAAGTTCCTATTCTCTAGAAAGTATAGGAACTTCGAAGTTCCCGCCAGCCTCGCAGAGCAG
GATTCCCGTTGAGCACCGCCAGGTGCGAATAAGGACAGTGAAGAAGGAACACCCGCTCGCGGGTGGGCCTAC
TTCACCTATCCTGCCCGGCTGACGCCGTTGGATACACCAAGGAAAGTCTACACGAACCCTTTGGCAAAATCCT
GTATATCGTGCGAAAAAGGATGGATATACCGAAAAAATCGCTATAATGACCCCGAAGCAGGGTTATGCAGCGG
AAAATGCAGCTCACGGTAACTGATGCCGTATTTGCAGTACCAGCGTACGGCCCACAGAATGATGTCACGCTGA
AAATGCCGGCCTTTGAATGGGTTCATGTGCAGCTCCATAAGCAAAAGGGGATGATAAGTTTATCACCACCGAC
TATTTGCAACAGTGCCGTTGATCGTGCTATGATCGACTGATGTCATCAGCGGTGGAGTGCAATGTCGTGACAC
AAGAATCCCTGTTACTTCTCGACCGTATTGATTCGGATGATTCCTACGCGAGCCTGCGGAACGACCAGGAATT
CTGGGAGCCGCTGGCCCGCCGAGCCCTGGAGGAGCTCGGGCTGCCGGTGCCGCCGGTGCTGCGGGTGCCCGGC
GAGAGCACCAACCCCGTACTGGTCGGCGAGCCCGGCCCGGTGATCAAGCTGTTCGGCGAGCACTGGTGCGGTC
CGGAGAGCCTCGCGTCGGAGTCGGAGGCGTACGCGGTCCTGGCGGACGCCCCGGTGCCGGTGCCCCGCCTCCT
CGGCCGCGGCGAGCTGCGGCCCGGCACCGGAGCCTGGCCGTGGCCCTACCTGGTGATGAGCCGGATGACCGGC
ACCACCTGGCGGTCCGCGATGGACGGCACGACCGACCGGAACGCGCTGCTCGCCCTGGCCCGCGAACTCGGCC
GGGTGCTCGGCCGGCTGCACAGGGTGCCGCTGACCGGGAACACCGTGCTCACCCCCCATTCCGAGGTCTTCCC
GGAACTGCTGCGGGAACGCCGCGCGGCGACCGTCGAGGACCACCGCGGGTGGGGCTACCTCTCGCCCCGGCTG
CTGGACCGCCTGGAGGACTGGCTGCCGGACGTGGACACGCTGCTGGCCGGCCGCGAACCCCGGTTCGTCCACG
GCGACCTGCACGGGACCAACATCTTCGTGGACCTGGCCGCGACCGAGGTCACCGGGATCGTCGACTTCACCGA
CGTCTATGCGGGAGACTCCCGCTACAGCCTGGTGCAACTGCATCTCAACGCCTTCCGGGGCGACCGCGAGATC
CTGGCCGCGCTGCTCGACGGGGCGCAGTGGAAGCGGACCGAGGACTTCGCCCGCGAACTGCTCGCCTTCACCT
TCCTGCACGACTTCGAGGTGTTCGAGGAGACCCCGCTGGATCTCTCCGGCTTCACCGATCCGGAGGAACTGGC
GCAGTTCCTCTGGGGGCCGCCGGACACCGCCCCCGGCGCCTGAAGTTCCTATTCCGAAGTTCCTATTCTCTAG
AAAGTATAGGAACTTCGAAGCAGCTCCAGCCTACACACGTCGCCCGTGCCTTCGGTTTCCACATGGTGTACCT
GTACTCGCGCAACGAGCACGTGCCGCCCGAGGTCGTACGCCACTTCCGCAAGGGGCTCGGCCCCGACCAGGTG
CTCTTCGTGAGCGGCAACGTCCGCTCCGGGCGGCAGGTCACCGAGTACCTCGACAGCGGGGCGGACTACGTGG
GGTTCGCCGGAGCCCTGGAACAGCCGGACTGGCGGTCCGCCCTCGCCGAGATCGCCGGGAGGCGGCCCGCCGC
CCCGGCCCGTCCGGGGAGCGGGCGGTGACGGCCGCCCCGGCCACGGCCCTGCCGGCGGACTACACGGAGACCA
TGCTCGCCGCCGAGGCCGCCAACCGCGACCATGTCACGCGGTGCGTCGCGCAGACCGGTGGGTCGCCGGACCT
GGTGGCGCACACCGCCGCCCTGCGCCTGTACCTGAGGGTGCCCCACTTCCTCACCGAGTGGACGACCGACCCG
GACCGGCGGGCCGCGGTGTCCCGCGCGCTGGCCCTCGACATCGTCTCCATGAAGCTCCTCGACGACCTGATGG
ACGACGACACCGGACTCGACCGGGTCGAACTCGCCTGTGTCTGCCTCCGCCTCCACCTGCGGGCGCTGCACGA
ACTGGAATCCCTCGCCCGGGACCCCAAGGCGGTGACGGACATCCTGGAGCAGGACGCCGTCCACCTCTGCGGC
GGCCAGATACGCACCAAACGCTCTCGGGCGACGAACCTCCGGGAGTGGCGCGCCCATGCGAGCACCTACGGCT
CCACCTTCCTGGGCCGCTACGGGGCACTCGCGGCCGCCTGCGGGGGGGAAGGCCAACCGGCGGACTCCGTAAG
GGAGTTCGCAGAGGCTTTCGCCATGACCATCACCATGGCGGACGACCTGACCGACTACGACCGCAACGGCGAG
CGGGACGGCAACCTCGCCCATCTGATGCGGACCGGGGCCGTGGCCGGCCAGGACGTCGTGGACCTGCTGGAGG
```

Figure S2. Knockout of *moeO5*. The genetic organization of *moeX5-moeN5* locus of *moe* gene cluster of wild type strain ATCC14672 (**a**) and that of Δ*moeO5*::*hyg* (**b**). Thick black arrows mark primers used to sequence the Δ*moeO5*::*hyg* locus amplified from dO5 strain. (**c**). Different background colors of the sequence corresponds to different parts of the deletion locus, as depicted in part **b**.

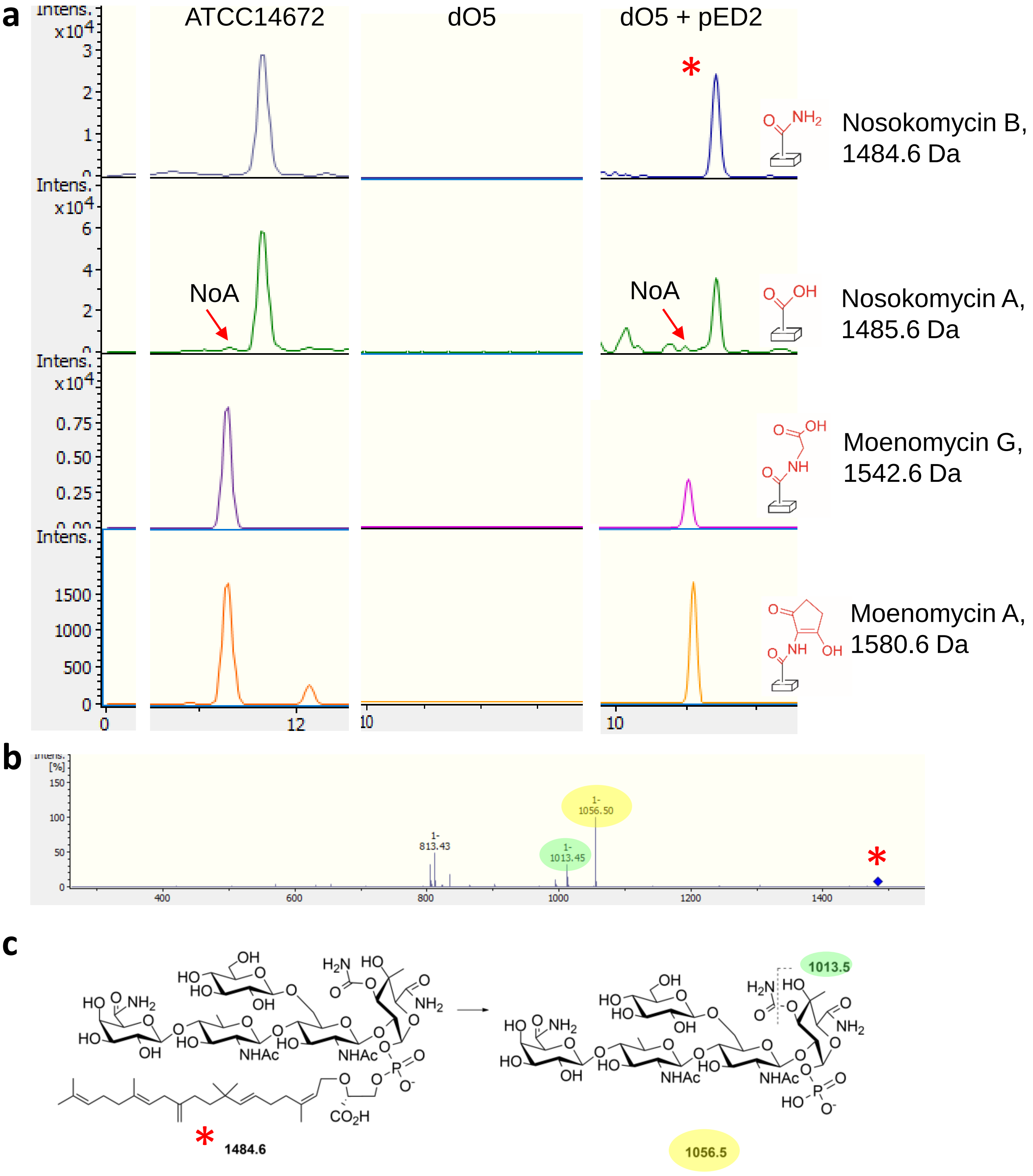

Figure S3. Mutant dO5 does not produce moenomycins. Extracted ion chromatogram (EIC) traces (**a**) for four final products of moenomycin biosynthetic pathway. Extracts from the parental strain (ATCC14672), the mutant (dO5) and the complemented mutant (dO5+pED2) were analyzed. The common lipid-pentasaccharide part of all moenomycins is shown as black prism. MS-MS analysis of major 1484.6 Da fraction (red asterisk) observed in dO5+pED2 extract (**b**) confirms its nosokomycin B identity (**c**).

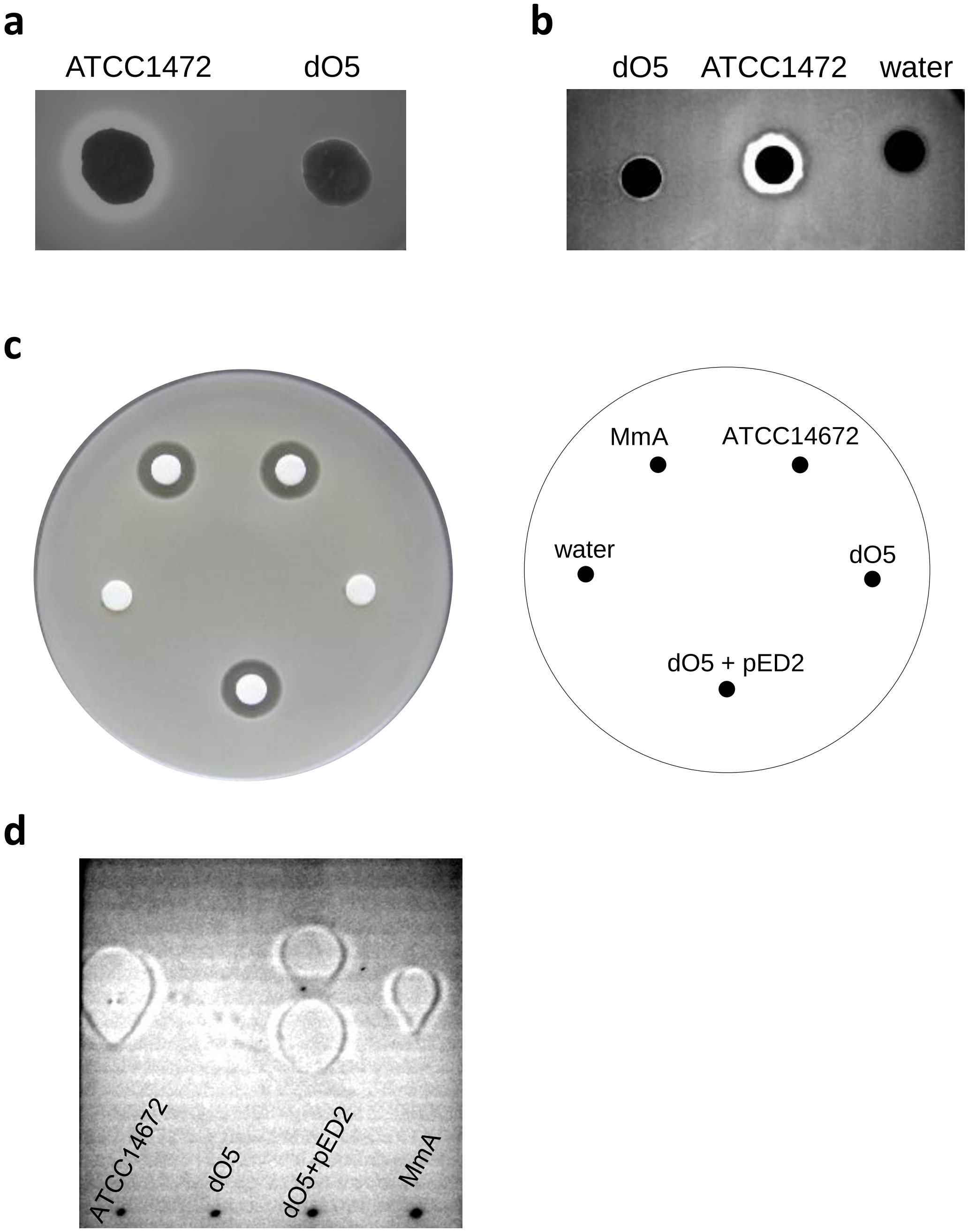

Figure S4. Bioassays of *S. viridosporus* strains as a facile readout of moenomycin production. *S. viridosporus* ATCC14672 and dO5 strains (104 CFU) were spotted onto TSA and grown for 5 days, then overlaid with moenomycin-susceptible culture *B. cereus* ATCC19637 (**a**). There is clearly visible halo of *B. cereus* growth inhibition by ATCC14672, although dO5 also displays some residual antibiotic activity, due to production of the other, unrelated to moenomycins, classes of antibiotics (2-4, see Supplementary references in the end of ESM). The whole methanol extracts of dO5, prior to C18 SPE (see Methods on the main) also displays antibiotic activity (**b**). The C18 SPE-purified extract of dO5 has no antibiotic activity in disc diffusion assay (**c**), and no moenomycin-specific bioactive spots on the biochromatogram (**d**). The extracts applied to the disc correspond to the equal amounts of the biomass collected from 5 mL of the fermentation broth. Pure moenomycin A (MmA, 3 µM) was used as a reference. Uncropped unedited versions of the images shown in this Figure are given in Fig. S11

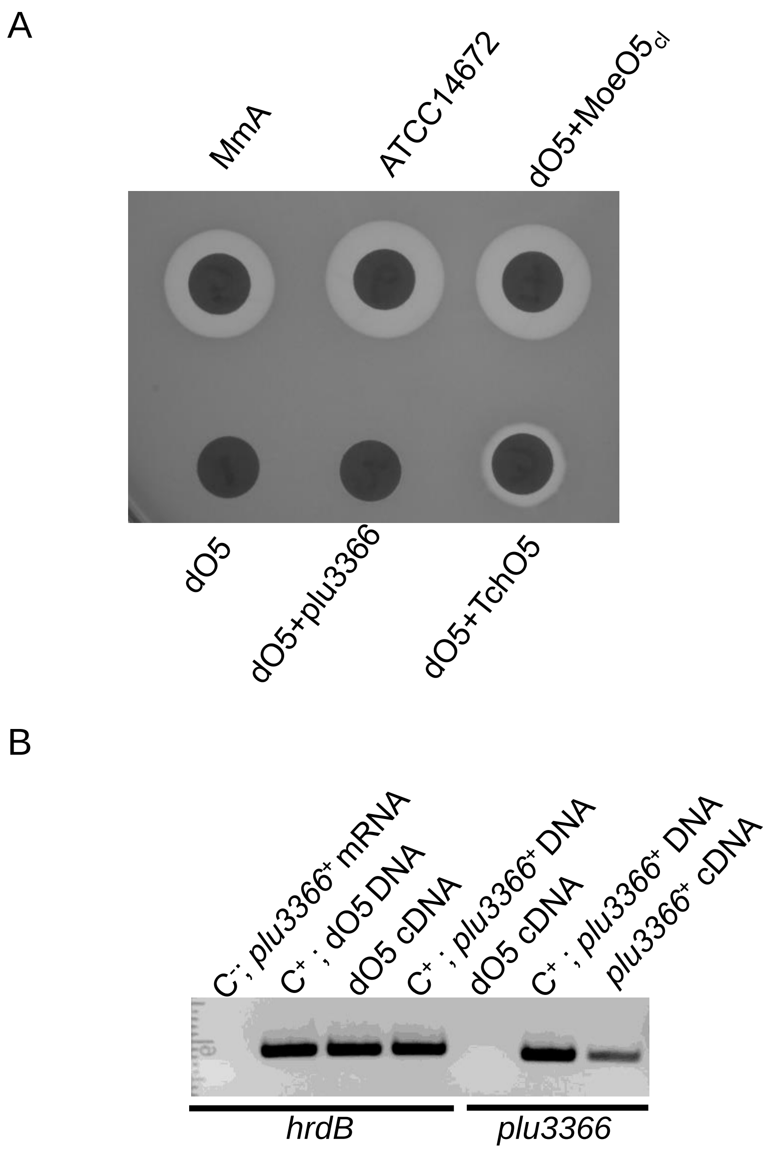

Figure S5. Disc diffusion assay of *S. viridosporus* dO5 strains expressing *moeO5* homologs (**A**) and RT-PCR analysis of *plu3366* transcription in dO5 strain (**B**). **A**, C18 SPE-purified methanol were used in this experiment (conditions, see Methods and legend to Fig. S4). MmA, 3 μM. **B**, the *plu3366* expression was analyzed in 48-h cultures; 100 and 200 ng of RNA sample were used per reaction (both for *plu3366*-plus strain and its parent dO5). Lower band in each lane represents positive control (*hrdB*). C- , negative control (RNA without RT step). Uncropped version of the gel image is shown in Fig. S12.

A

Gene Name	name	Organism	Protein ID
plu3366	Photo3	Photorhabdus laumondii subsp. laumondii (strain DSM 15139 / CIP 105565 /TT01)	CAE15740.1
SCLAV_p1286	S. clav	Streptomyces clavuligerus.	EFG04772.2
PAU_01282	Photo2	Photorhabdus asymbiotica subsp. asymbiotica (strain ATCC 43949 / 3105-77)	CAQ83374.1
GCM10018772_35140	S. fumanus	Streptomyces fumanus.	GHF07148.1
GCM10010274_29650	S. lavendo	Streptomyces lavendofoliae.	GGU40040.1
GCM10010123_40620	Pilimelia	Pilimelia anulata.	GGK06725.1
FOE67_01540	S. caldi	Streptomyces calidiresistens.	MBB0228227.1
JEQ17_04745	S.lilifuscus	Streptomyces liliifuscus.	QQM38848.1
GPY51_17110	Photo	Photorhabdus laumondii subsp. laumondii	NDL40436.1
GTY54_10075	S.sp SID625	Streptomyces sp. SID625.	MYR56571.1
JD77_04767	Micro_oliva	Micromonospora olivasterospora.	TWH69753.1
DMH12_03700	S. WAC04229	Streptomyces sp. WAC 04229.	RSN64137.1
CKY02_05135	Photo1	Photorhabdus bodei.	RAX13825.1
DKG34_16210	S. sp. NWU49	Streptomyces sp. NWU49.	PWJ06421.1
B5D80_04885	Micro_wench	Micromonospora wenchangensis.	OWV11064.1
AV521_05130	S. IMTB2501	Streptomyces sp. IMTB 2501.	OLZ73696.1
VSP9026_03496	Vibrio	Vibrio spartinae.	SIO95744.1
GA0070216_108134	Micromonosp.	Micromonospora matsumotoense.	SCF27734.1
SLINC_6335	S. lincoln	Streptomyces lincolnensis.	ANS68559.1
FHX34_104997	Actinoplanes1	Actinoplanes teichomyceticus.	ANQ31710.1|TWG14691.1
A8713_30305	S. sp. SAT1	Streptomyces sp. SAT1.	ANH95778.1
A4U61_00760	Strep-H-KF-8	Streptomyces sp. H-KF8.	OBQ54362.1
ADK41_32265	S. caelestis	Streptomyces caelestis.	KOT30539.1
CF54_14045	S. Tu6176	Streptomyces sp. Tu 6176.	EYT82319.1
moeO5	ATCC14672	Streptomyces viridosporus.	ABJ90164.1

B

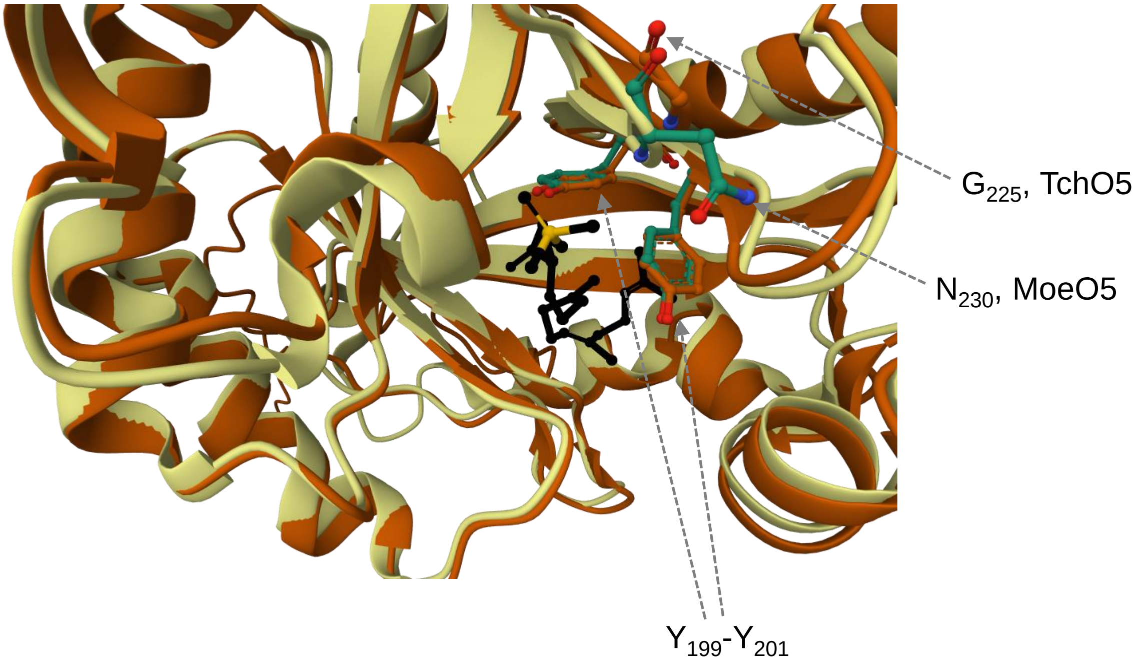

Figure S6. Part **A**, proteins used to build Plu3366 SSN (Fig. 3, main text). Part **B**, MoeO5-TchO5 superposition. MoeO5 is light yellow, TchO5 is brown, MoeO5 ligand, FsPP, is shown as black ball and stick model, phosphorus atom is orange. Tyr199-Tyr201 motif has the same spatial location in both proteins (shown as balls and sticks). Gly225 in TchO5 occupies the position that harbors Asn in MoeO5, according to this model. Analogous situation is observed in Plu3366, except that it carries distal Asn residue.

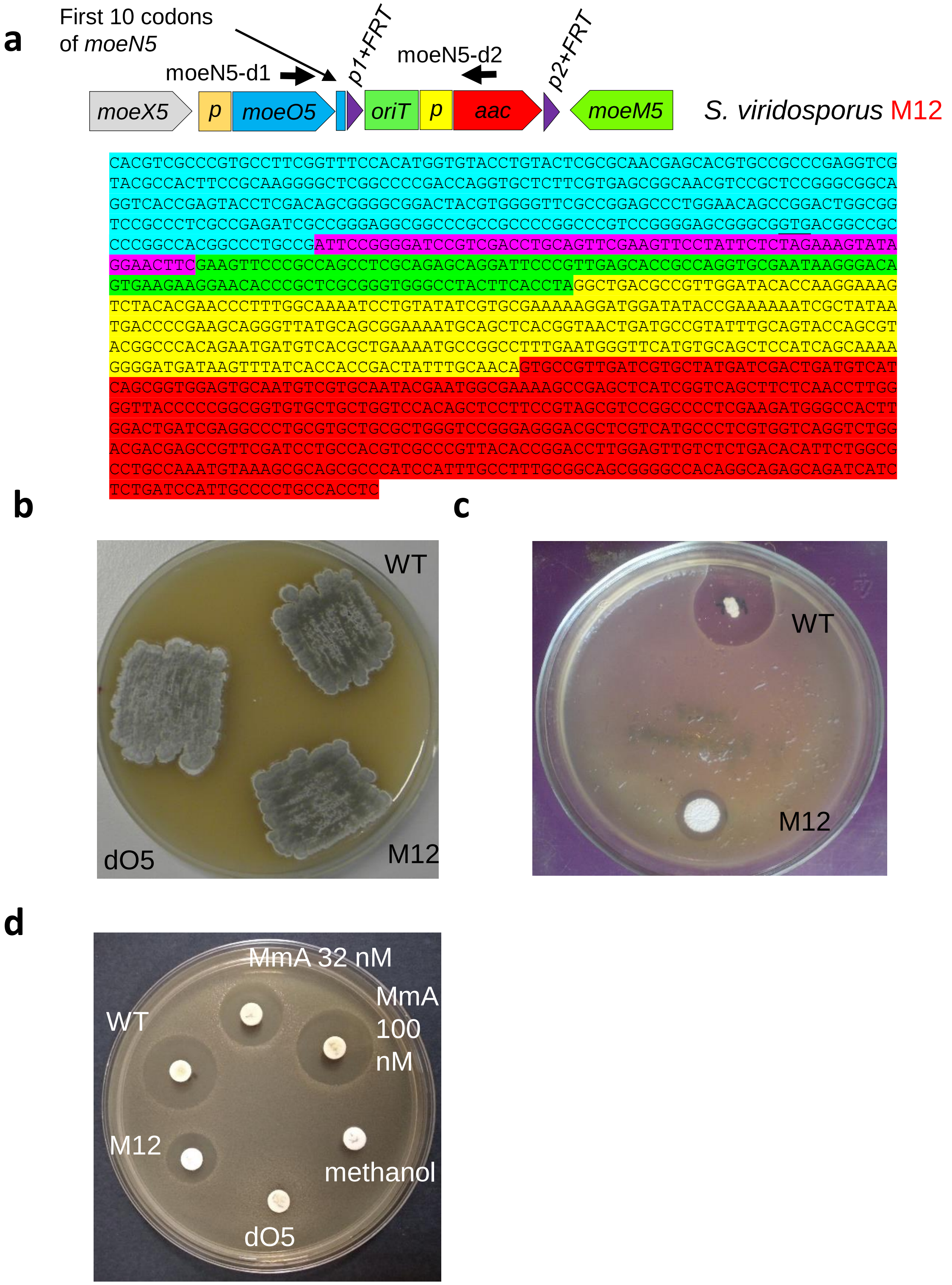

Figure S7. The *moeN5*-null mutant *S. viridosporus* M12: genotype (**a**) and phenotype (**b**-**e**). Part **b**, 5-day lawns of the strains. In part **c** the strains were spotted onto TSA and grown for 5 days, then overlaid with soft agar containing MmA sensitive strain *B. cereus* ATCC19637. Disc diffusion assay (**d**) of the C18 SPE-purified moenomycins were carried out as described in legends to Fig. S4.

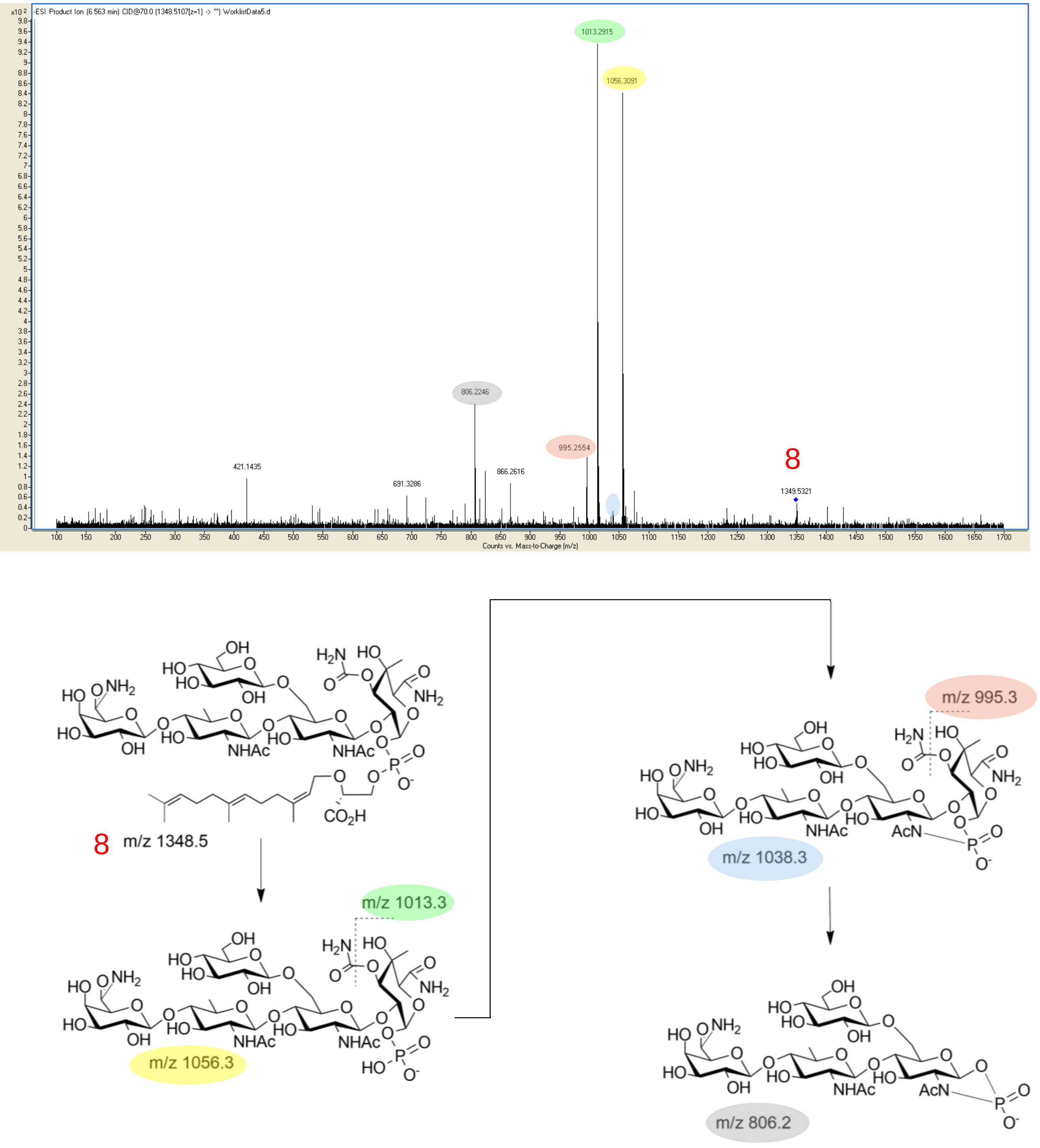

Figure S8. Collision-induced dissociation spectra for the compound **8**.

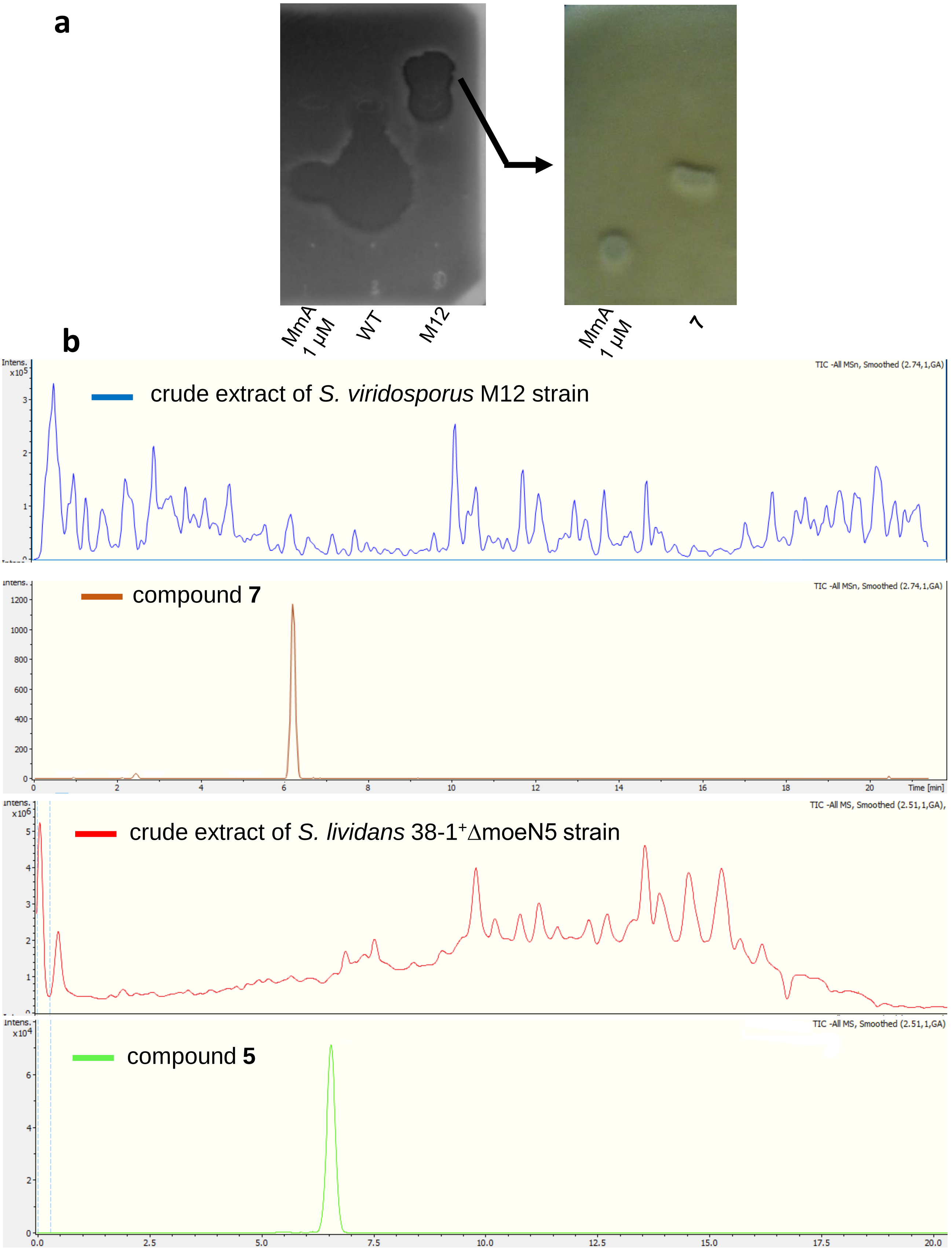

Figure S9. Purification of compounds **5** and **7** for bioassays. C18 SPE-purified extract of M12 was used to isolate compound **7** using TLC-based method as described in main text, giving single bioactive spot (**a**). Total ion chromatograms of crude extracts and purified compounds **7** and **5** (1 μM) are shown in part **b**. MS-MS spectrum of compound **7** - see in Fig. S9, **5** – ref. [5].

a

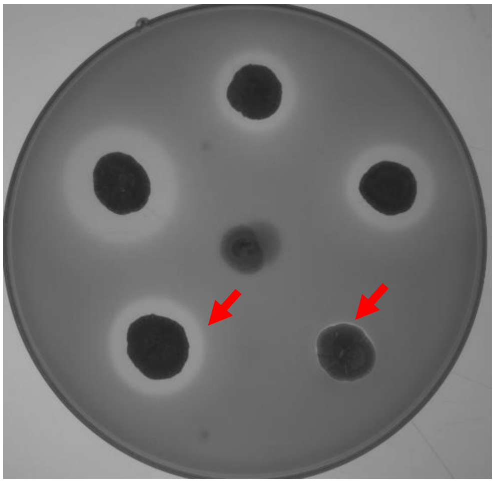

b

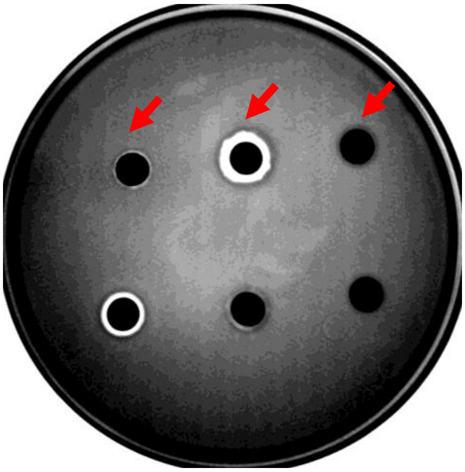

d

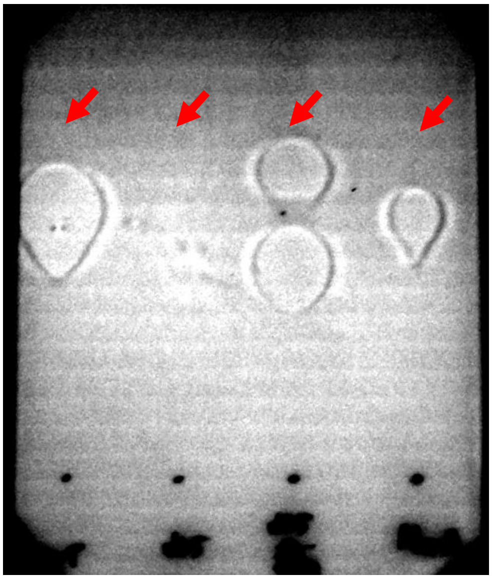

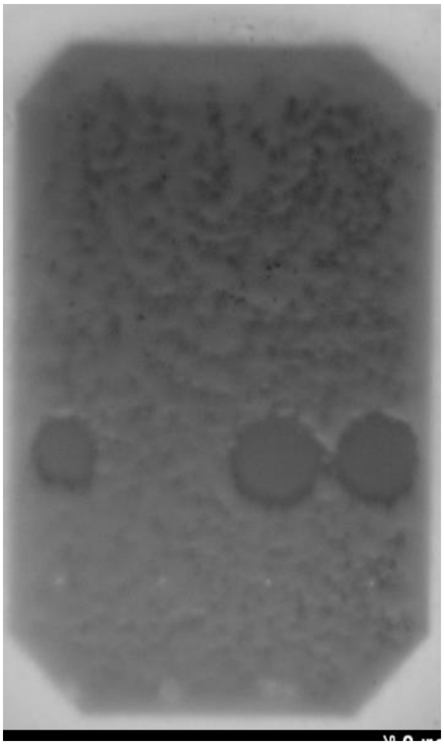

Figure S10. Uncropped unedited versions of the images shown in Fig. S4. Part S11a corresponds to Fig S4a; S11b – S4b; S11d – S4d. Red arrows mark the elements shown in Fig. S4. The rest of the images correspond to samples/ bioassays unrelated to this manuscript and thus were cropped in Fig. S4

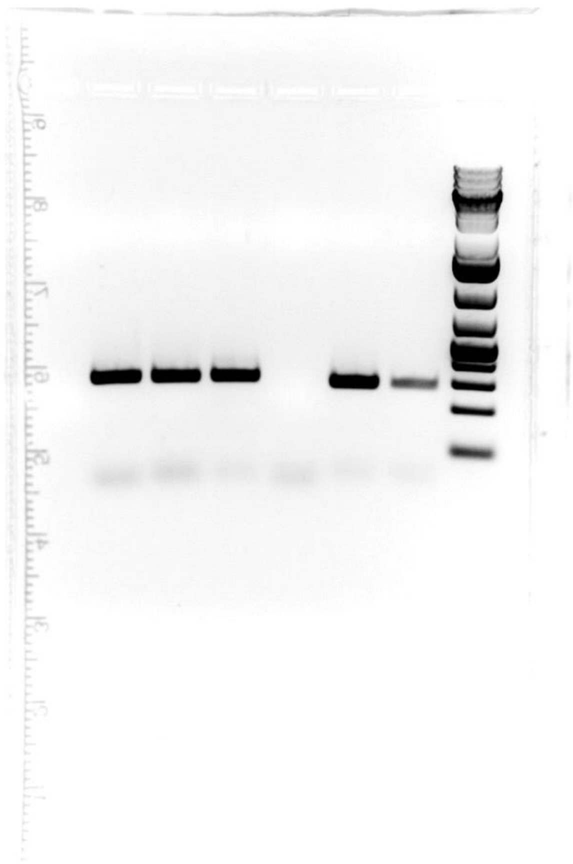

Figure S11. Uncropped version of the gel image shown in Fig. S5b.

Supplementary References

1. Ren F, Ko TP, Feng X, Huang CH, Chan HC, Hu Y, Wang K, Ma Y, Liang PH, Wang AH, Oldfield E, Guo RT. Insights into the mechanism of the antibiotic-synthesizing enzyme MoeO5 from crystal structures of different complexes. Angew Chem Int Ed Engl. 2012;51:4157–60
2. Zhang C, Seyedsayamdost MR. Discovery of a Cryptic Depsipeptide from Streptomyces ghanaensis via MALDI-MS-Guided High-Throughput Elicitor Screening. Angew Chem Int Ed Engl. 2020 Dec 14;59(51):23005-23009. doi: 10.1002/anie.202009611.
3. Makitrynskyy R, Tsypik O, Nuzzo D, Paululat T, Zechel DL, Bechthold A. Secondary nucleotide messenger c-di-GMP exerts a global control on natural product biosynthesis in streptomycetes. Nucleic Acids Res. 2020 Feb 20;48(3):1583-1598. doi: 10.1093/nar/gkz1220.
4. Makitrynskyy R, Keller L, Kaur A, Tsypik O, Munz L, Bechthold A, Müller R. Olikomycin A-A Novel Calcium-Dependent Lipopeptide with Antibiotic Activity Against Multidrug-Resistant Bacteria. Chemistry. 2025 Feb 6;31(8):e202403985. doi: 10.1002/chem.202403985.
5. Ostash, B., Doud, E. H., Lin, C., Ostash, I., Perlstein, D. L., Fuse, S., Wolpert, M., Kahne, D., & Walker, S. (2009). Complete characterization of the seventeen step moenomycin biosynthetic pathway. *Biochemistry*, *48*(37), 8830–8841. https://doi.org/10.1021/bi901018q
